# Enhanced features of *Dictyoglomus turgidum* Cellulase A engineered with carbohydrate binding module 11 from *Clostridium thermocellum*

**DOI:** 10.1038/s41598-018-22769-w

**Published:** 2018-03-13

**Authors:** Chiara Cattaneo, Patrizia Cesaro, Stefano Spertino, Sara Icardi, Maria Cavaletto

**Affiliations:** 1 University of Piemonte Orientale, Dipartimento di Scienze e Innovazione Tecnologica Complesso Universitario S. Giuseppe, Piazza S. Eusebio 5, Vercelli, 13100 Italy; 2 University of Piemonte Orientale, Dipartimento di Scienze e Innovazione Tecnologica Viale T. Michel 11, Alessandria, 15121 Italy

## Abstract

Lignocellulosic biomass (LCB) is a low-cost and abundant source of fermentable sugars. Enzymatic hydrolysis is one of the main ways to obtain sugars from biomass, but most of the polysaccharide-degrading enzymes are poorly efficient on LCB and cellulases with higher performances are required. In this study, we designed a chimeric protein by adding the carbohydrate binding module (CBM) of the cellulosomal enzyme *Ct*Lic26A-Cel5E (endoglucanase H or CelH) from *Clostridium (Ruminiclostridium) thermocellum* to the C-terminus of Dtur CelA, an interesting hyperthermostable endoglucanase from *Dictyoglomus turgidum*. The activity and binding rate of both native and chimeric enzyme were evaluated on soluble and insoluble polysaccharides. The addition of a CBM resulted in a cellulase with enhanced stability at extreme pHs, higher affinity and activity on insoluble cellulose.

## Introduction

Lignocellulosic biomass (LCB) represents an invaluable source of “green energy” providing fermentable sugars for different purposes, among which the production of bioethanol. However, the complete exploitation of LCB is still far from being accomplished, due to LCB complexity and recalcitrance, and several efforts are required to improve the efficiency of the process and to increase the product yield. Enzymatic hydrolysis is one of the main ways to obtain sugars from biomass, but often the polysaccharide-degrading enzymes are poorly efficient on LCB. Therefore, the characterization (or engineering) of cellulases with higher performances becomes mandatory to keep LCB sugar production at competitive prices if compared with sugars obtained from readily degradable biomass. *Dictyoglomus turgidum* is an extreme thermophilic anaerobic Gram-negative bacterium that produces a broad range of Carbohydrate Active enZYmes (CAZymes)^1–3^. Currently (January 2018) the CAZy database^4^ (http://www.cazy.org) reports 57 glycoside hydrolases (GHs) in *D*. *turgidum* genome. The *Dtur*_0670 gene codes for a protein of 312 amino acids belonging to the GH5 family, subfamily 25 (GH5_25)^5^, named Cellulase A (UniProtKB: B8DZM2). Dtur CelA is a hypertermostable endoglucanase showing both endo- and exo-activity on soluble and insoluble β-(1,4)-linked glucose-containing substrates as well as endo-activity on soluble and insoluble β-(1,4)-linked mannose containing substrates, but does not recognize β−1,3-linked glucose residues^6^. Most cellulases contain non-catalytic carbohydrate-binding modules (CBMs) connected to the catalytic domain (CD) through a linker peptide that is sometimes highly flexible^7^. The CBMs bring the CD close to the target substrate, increasing the rate of catalysis, although the mechanism by which this interaction occurs is not completely clear^7,8^. It is known that CBMs recognize different polysaccharides in a specific way, thus the CBMs present in cellulases bind cellulose, enhancing enzyme activity on the insoluble polysaccharide^9,10^. According to CAZy, CBMs are classified in 83 families and are grouped into three types, A, B and C, depending upon their ligand-binding site interaction. Type A CBMs bind the flat surface of crystalline polysaccharides, Type B CBMs bind internally on glycan chains and Type C CBMs bind the termini of glycans (“small sugar-binding”)^7,11^. *Clostridium thermocellum* (now reclassified as *Ruminiclostridium thermocellum*) is a thermophilic, anaerobic, Gram-positive bacterium able to grow on crystalline cellulose^12^. In *C*. *thermocellum*, the degradation of cellulose is carried out by a high molecular weight complex, the cellulosome, consisting of a number of enzymes, each one containing a dockerin domain, which binds to a cohesin domain, enabling the assembly of several enzymes to be brought together into the cellulosome^13^. *C*. *thermocellum* Lic26A-Cel5E (also known as endoglucanase H or CelH; UniprotKB: P16218) is a cellulosomal enzyme containing two catalytic modules (Lic26A and Cel5E), a CBM11 and two C-terminal type I dockerin domains. Lic26A is a GH26 hydrolase with β1,3–1,4-mixed linked endoglucanase activity, while Cel5E is a bifunctional β-1,4-endoglucanase/xylanase classified as GH5_25^5,14^. *Ct*CBM11 (type B CBM) accommodates a single polysaccharide chain in its cleft^15,16^, and presents a β-sandwich with a classical distorted β-jelly roll fold, where two anti-parallel β-sheets form a convex and a concave side, where the glycan binding takes place^17^. *D*. *turgidum* CelA has no electronically detectable CBM, as previously reported^6^, thus we designed a chimeric protein by adding the *Ct*CBM11 to the C-terminus of Dtur CelA catalytic domain, connecting them by a linker peptide from *Ct*Lic26A-Cel5E. The aim of this study was to evaluate the effects of protein engineering on the activity and binding rate of Dtur CelA on both soluble and insoluble polysaccharides.

## Results and Discussion

### Expression and purification of native and chimeric Dtur CelA

**Figure 1:**
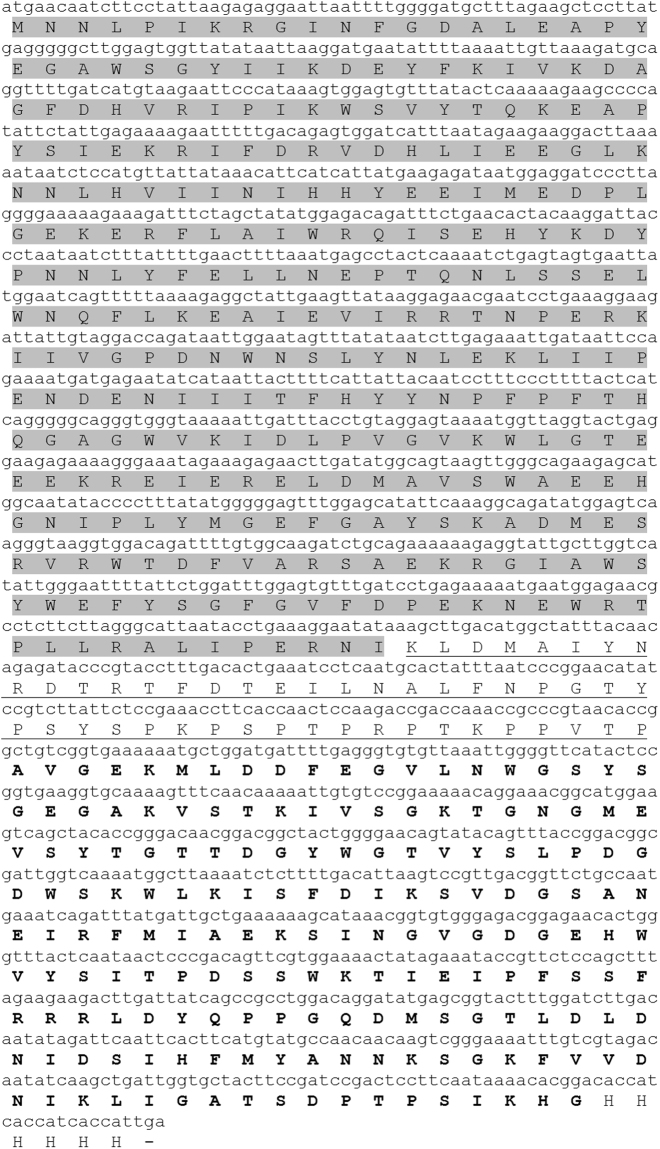
Nucleotide and amino acid sequence of chimeric Dtur CelA. The amino acid sequence of Dtur CelA is highlighted in gray, the linker sequence of *Ct*CelH is underlined, and *Ct*CBM11 is in bold.

**Figure 2:**
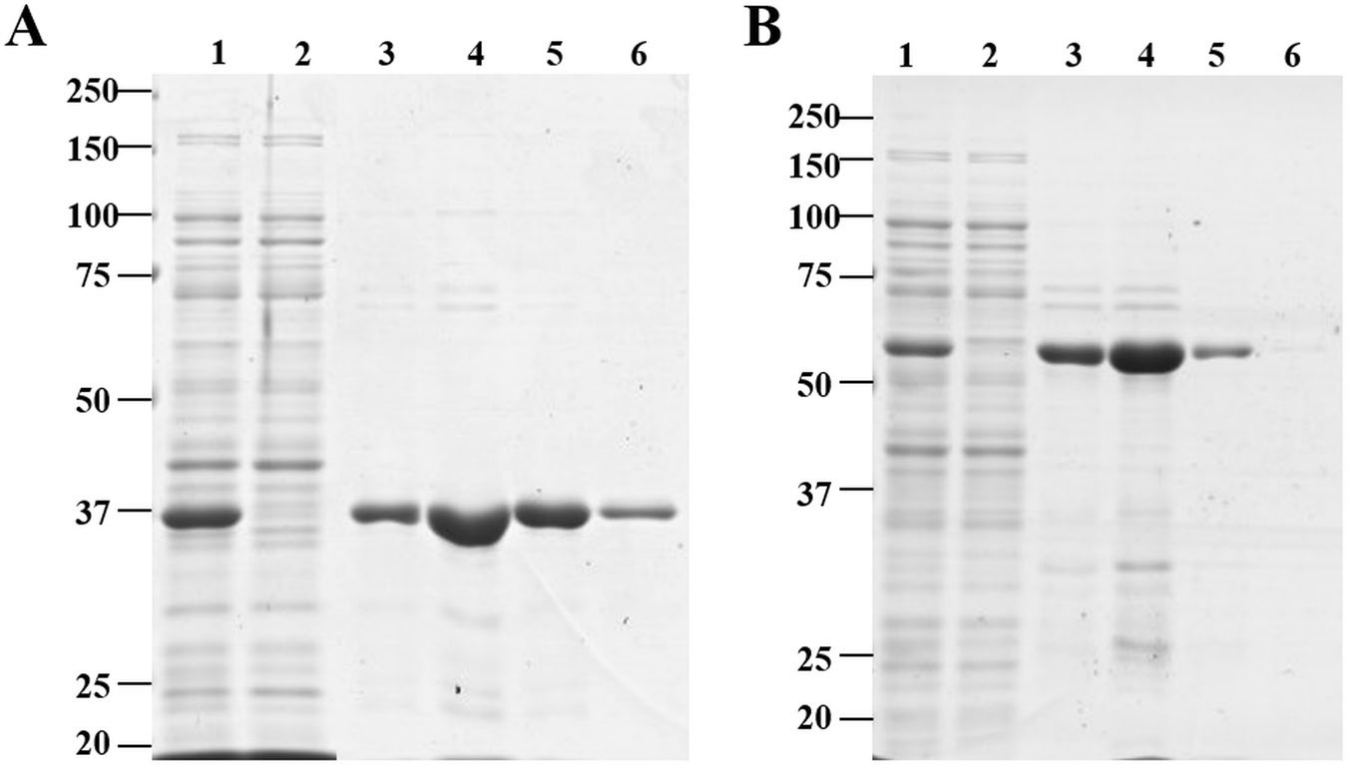
SDS-PAGE of native (**A**) and chimeric (**B**) Dtur CelA after purification by affinity chromatography. Lane 1: total protein extract; lane 2: flow through; lanes 3–6: elution fractions. The gels were Coomassie stained. Molecular weight markers are indicated on the left.

We designed a chimeric protein by adding the CBM11 and linker peptide from *Ct*CelH to the C-terminus of Dtur CelA. CBM 11 from *Clostridium thermocellum* has been chosen, since it is naturally linked to a catalytic domain (Cel5E) classified as GH5 in the cellulosomal enzyme CelH. The amino acid sequence of chimeric Dtur CelA is reported in Fig. 1. The vector pET20b-CelA codes for a protein with theoretical molecular weight (MW) of 37.8 kDa and isoelectric point (pI) 5.49, while pET20b-CelA-linker-CBM11 codes for a protein with theoretical MW of 62.7 kDa and pI 5.42. Both the recombinant proteins were produced in soluble form. The protein profile after the purification step is shown in Fig. 2 and S1 (supplementary material). The molecular weights estimated by SDS-PAGE were around 37 kDa for Dtur CelA and 63 kDa for chimeric Dtur CelA, in agreement with those predicted from their amino acid sequence. After the removal of imidazole and other buffer components by dialysis, the addition of 20% glycerol was found to be the best preserving of protein function.

### 2-DE profile of native and modified Dtur CelA

**Figure 3:**
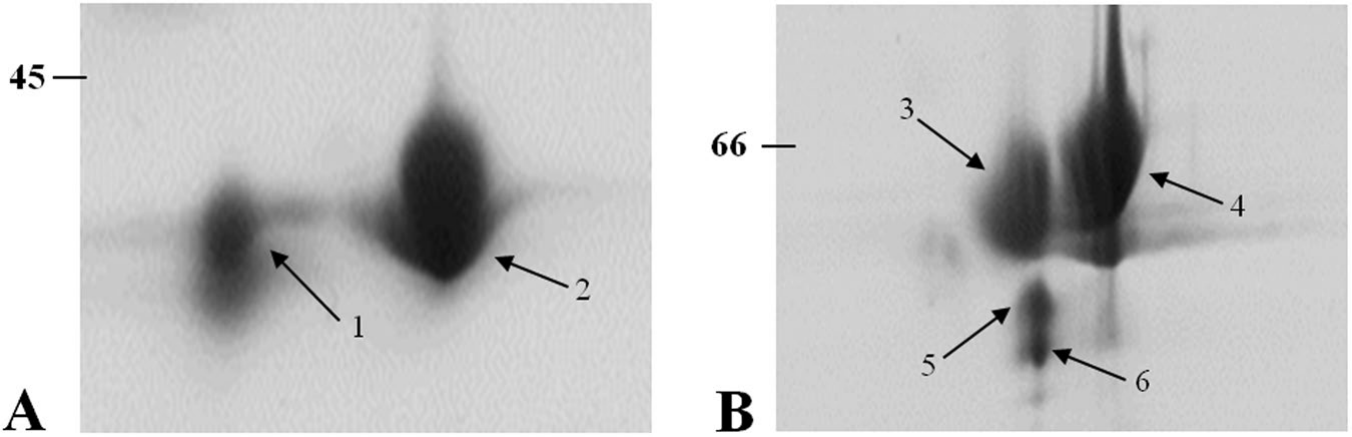
2-DE separation of Dtur CelA (**A**) and chimeric Dtur CelA (**B**). 75 μg of protein were loaded and focused on pH 3–10 IPG-strip before separation on a 10% (**A**) and 12% (**B**) polyacrylamide gel. Molecular weight markers are indicated on the left.

Dtur CelA migrates as two spots with the same MW but with different pI (spots 1 and 2, Fig. 3A). These spots were excised from the gel, trypsin digested and submitted to LC-MS/MS analysis. Spots 1 and 2 were identified as Glycoside hydrolase 5 from *D*. *turgidum* (gi|217967063/ YP_002352569.1), whose sequence coverage is reported in Figures S2 and S3 (supplementary material). Chimeric Dtur CelA migrates as four main spots (3, 4, 5, 6, Fig. 3B), identified as Glycoside hydrolase 5 from *D*. *turgidum* (gi|217967063/ YP_002352569.1) and Carbohydrate-binding module family 11 protein of CelH from *C*. *thermocellum* (gi|125973984/ YP_001037894.1, now annotated as endoglucanase WP_011838089.1 which represents the whole CelH protein) with the sequence coverages reported in Figures S4, S5, S6 and S7 (supplementary material). MS/MS analysis confirmed the presence of the entire *Ct*CBM11 domain in the final protein product. The identified peptides are highlighted in bold red. It is very likely that both proteins Dtur CelA and chimeric Dtur CelA migrate as multiple spots for side reactions due to the iodoacetamide used in sample preparation^18^.

### Biochemical properties and kinetic parameters

**Figure 4:**
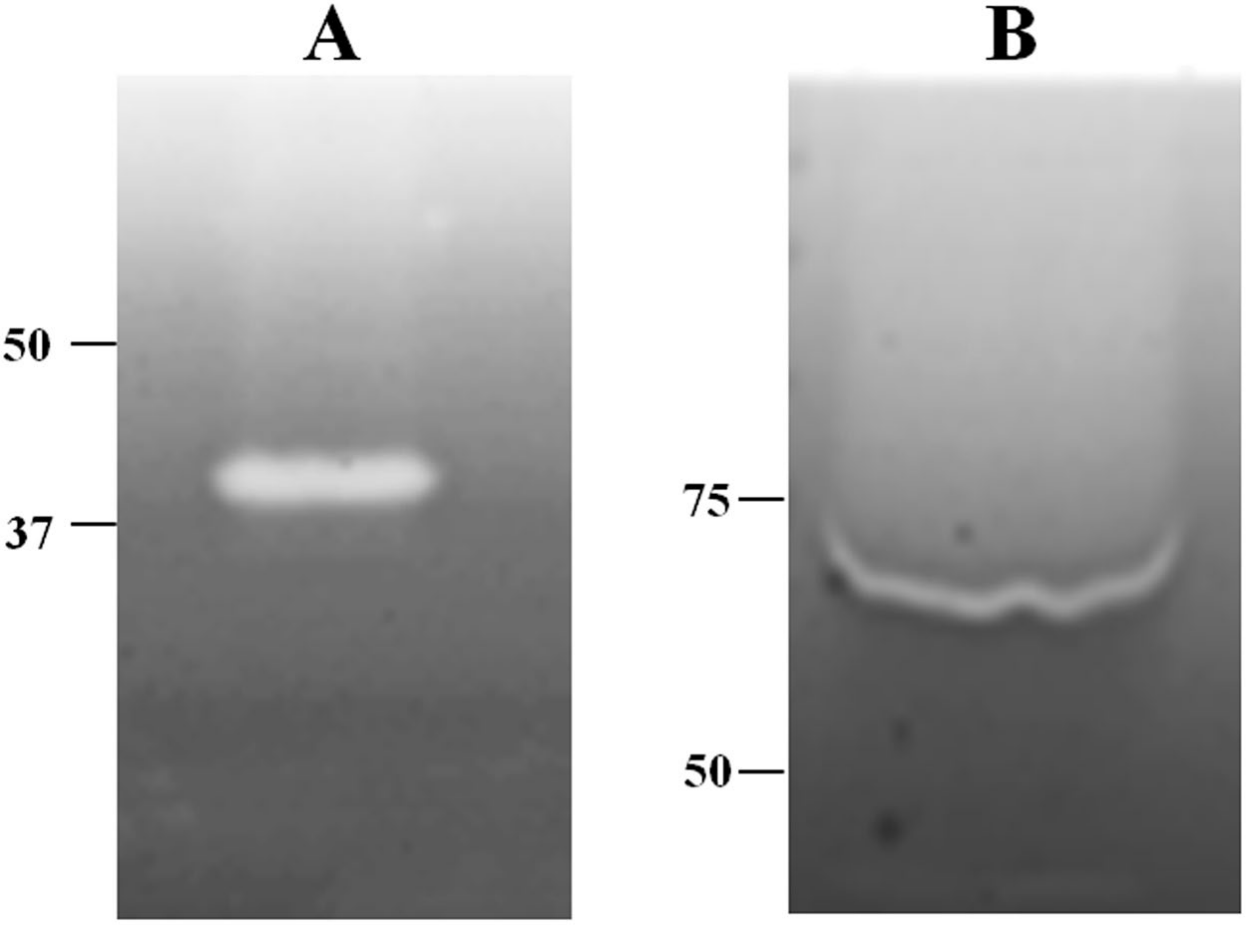
Zymography on 0.4% AZO-CMC of native (**A**) and chimeric (**B**) Dtur CelA. Molecular weight markers are indicated on the left.

**Figure 5:**
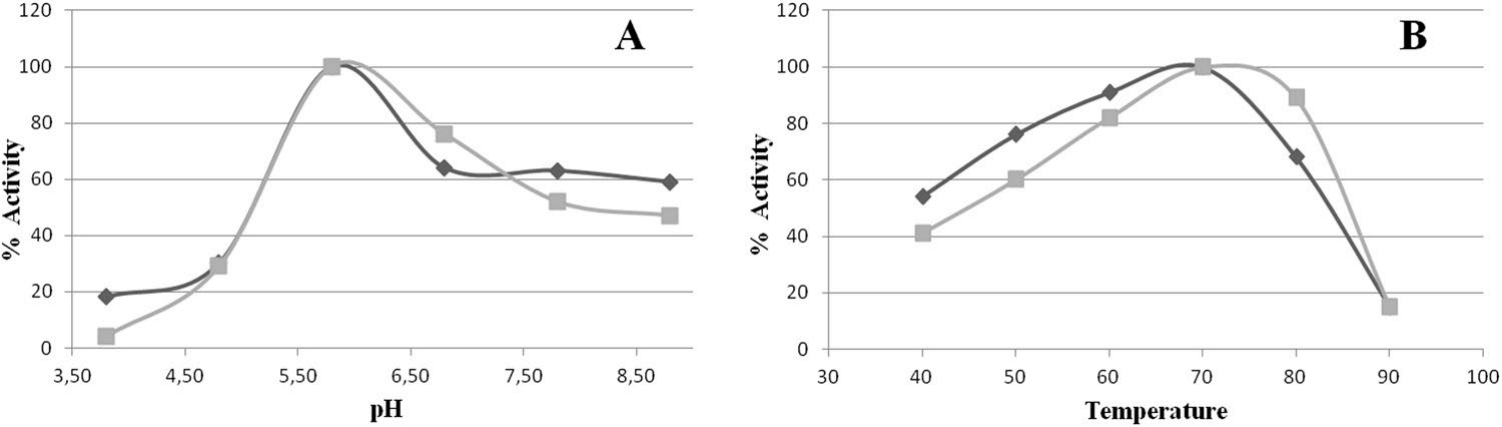
Optimal pH and temperature: percentages of activity observed on β-glucan for native (squares) and chimeric (diamonds) Dtur CelA in Na-acetate 50 mM buffer at different pHs (**A**) and temperatures (**B**).

Zymography on 0.4% AZO-CMC revealed endoglucanase activity of the two protein forms as a clear band over a dark background (Fig. 4 and S8 of supplementary material). Since *D*. *turgidum* and *C*. *thermocellum* are thermophilic bacteria, their proteins are likely to be thermo-resistant as well. In order to evaluate the effects of *Ct*CBM11 addition on the optimal pH and temperature of Dtur CelA, the two enzyme variants were assayed in the °T range of 40 °C–90 °C and pH range of 3.8–8.8. Optimal assay conditions of both proteins were 70 °C and pH 5.8 (Fig. 5). Chimeric Dtur CelA percentage of activity at 40 °C was 54%, then it increased up to 70 °C, where it reached its top. At 80 °C the activity decreased to 68% and dropped to 15% at 90 °C. Dtur CelA percentage of activity was lower at 40 °C (40%), reached 100% at 70 °C, then it slowly declined to 89% at 80 °C and sharply dropped to 15% at 90 °C. These data are in accordance with those reported previously: Dtur CelA temperature optimum was stated between 70 °C and 80 °C^6^. Concerning *Ct*CBM11, Viegas and collaborators observed that it does not change its structure at 25 °C and 50 °C, confirming its stability in this °T range; moreover, a closer contact between *Ct*CBM11 and cellohexaose was detected at 50 °C than 25 °C^19^. At the most acidic pH tested, chimeric Dtur CelA showed higher relative activity compared to the native form (20% vs 0%). The two enzymes showed a superimposable increase of activity in the pH range 4.8–5.8 (from 30% to 100%). At pH 6.8, relative activity of modified Dtur CelA was slightly lower than native Dtur CelA (64% vs 76%), while it retained higher activity in the pH range 7.8–8.8 when compared to Dtur CelA (60% vs 50%). The detected optimal pH of native Dtur CelA is in accordance with Brumm and collaborators^6^, who found a peak of activity between pH 5.8 and 6.8 for Dtur CelA. The addition of *Ct*CBM11 to Dtur CelA might have enhanced protein stability, as confirmed by the higher relative activity of chimeric Dtur CelA respect with Dtur CelA at extreme pH values, while conserving its optimum at pH 5.8. The enzyme stability over time at 4 °C was also monitored by assaying activity for nine months and at the end of this time period, the chimera did not present any loss of activity (Supplementary material S9, S10**)**.

Native and chimeric Dtur CelA showed K_m_ values of 1.6 × 10^−2^ and 5.6 × 10^−4^ mM, and V_max_ values of 19.65 and 1.87 mM min^−1^ respectively, using barley β-glucan as substrate. The catalytic efficiency (K_cat_/K_m_) of Dtur CelA and the chimera were 2.6 × 10^9^ M^−1^s^−1^ and 1.11 × 10^10^ M^−1^s^−1^, respectively Table 1). The addition of *Ct*CBM11 decreased the K_m_ of Dtur CelA of two orders of magnitude and increased its efficiency, with a consequent higher affinity and specificity of chimeric Dtur CelA for barley β-glucan. This is in agreement with previous studies, where it was observed the specificity of *Ct*CBM11 for β-(1,4)- and β-(1,3)- β-(1,4)- mixed sugars^17^.

**Table 1:** Michaelis-Menten parameters (K_m_, V_max_) and catalytic efficiency (K_cat_/K_m_) of native and chimeric Dtur CelA using low viscosity barley β-glucan as substrate.

Enzyme	K_m_ (mM)	V_max_ (mM min^−1^)	K_cat_/K_m_ (M^−1^ s^−1^)
Dtur CelA	1.6 × 10^–2^	19.65	2.6 × 10^9^
Chimeric Dtur CelA	5.6 × 10^–4^	1.87	1.11 × 10^10^

Enzyme activities on soluble and insoluble substrates are listed in Table 2. Dtur CelA engineering resulted in a chimeric enzyme which conserved catalytic activity. Native and chimeric Dtur CelA were more active on barley β-glucan, a highly accessible polysaccharide, than on the insoluble substrates AZCL-HE-Cellulose and Avicel. The activity of native Dtur CelA on barley β-glucan and AZCL-HE-Cellulose was higher than the chimeric form. On the contrary, the chimeric variant showed an increased activity on Avicel respect with native Dtur CelA. A similar trend was reported for *Thermotoga maritima* Cel5A and its chimeric forms CBM1-Cel5A and Cel5A-CBM6, where the native enzyme showed higher activity on soluble cellulose (CMC) and lower activity on insoluble cellulose (Avicel) in comparison with the two chimeric enzymes^20^.

**Table 2:** Enzyme activity on soluble and insoluble polysaccharidic substrates.

Enzyme	β-glucan	HE-cellulose	Avicel
Dtur CelA	31176 ± 3507	111.5 ± 0.3	1.85 ± 0.04
Chimeric Dtur CelA	9615 ± 502	64.5 ± 4.7	3.22 ± 0.11

Activity is expressed as µmol × min^−1^ per 1 µmol enzyme.

*Ct*CBM11 interacts with β−1,4-linked glucans, but shows a preference for β-1,3–1,4 mixed linked glucose polymers, with the possibility to bind only one β-1,3-linked glucose in its cleft^14^. The structure of barley β-glucan corresponds to the “requirements” of *Ct*CBM11, since its repetitive unit is composed by 2–3 consecutive β-1,4 linkages, separated by a β-1,3 linkage. This preference is confirmed by the lower Km observed for chimeric Dtur CelA compared with native Dtur CelA on barley β-glucan, displaying an enhanced affinity for this polymer exerted by the addition of *Ct*CBM11. These findings are in accordance with a very recent work^21^ on the *Bacillus subtilis* endo-β-1,4-glucanase BsCel5A (a GH5 with high activity on β-1,3–1,4-linked glucans), where the exchange of the CBM3, present in the wild type BsCel5A, for *Ct*CBM11 (with high affinity for β-1,3–1,4 glucans) increased the catalytic efficiency of the enzyme on such substrates.

However, despite the higher affinity for barley β-glucan, we did not observe an increase of activity of the chimeric enzyme for this substrate. This evidence is supported by other studies^22,23^, where higher enzyme activities on a specific substrate did not correlate with the binding amounts for that substrate. On the contrary, it seems that the highest activity is correlated with a moderate binding affinity of the CBM.

In our case, a possible explanation could be provided by the inability of Dtur CelA to recognize β-1,3 linkages: this fact may not represent a problem for the native enzyme, that could leave the oligomer when encountering a non-recognized bond but, in the chimeric enzyme, the tighter binding of CtCBM11 to barley β-glucan might impede a free movement of Dtur CelA along the chain, forcing its contact with an unrecognized bond. This is supported by previous observations^23^ that tight binding of a CBM may limit the number of productive hydrolytic events and the catalytic domain may not have an easy access to new glycosidic bonds.

The activity of chimeric Dtur CelA was almost half of that measured for native Dtur CelA on AZCL-HE-Cellulose. It is argued that the enhanced activity of CBM-containing cellulases on their substrates may be related to increased enzyme concentration onto the surface and penetration into the fibers of the cellulosic substrate^24,25^. Carvalho and coworkers^14^ detected a much weaker binding of *Ct*CBM11 to HE-cellulose when compared to barley β-glucan. Thus, the lower activity of the chimera on HE-cellulose may be due to the poor interaction between enzyme and substrate.

On the contrary, the chimeric enzyme doubled its activity on Avicel compared with the native form. The increase of activity on insoluble rather than soluble substrates is a common trait of many CBMs^11^. Other studies reported the ability of *Ct*CBM11 to increase the activity of GH5 catalytic domain of CtCelH against the insoluble substrate Avicel but not against soluble substrates such as lichenan and carboxymethyl cellulose (CMC)^22,26^, and the absence of CBM reduced the activity of CtCel5E on Avicel, ball-milled cellulose and oat-spelt xylan compared with its CBM containing forms (wild type or CBM3 and CBM6 chimeras)^22^.

Here we observed that the new introduction of a CBM (*Ct*CBM11) to an endoglucanase that does not present a CBM in its native form (DturCelA), could ameliorate its activity on insoluble cellulosic substrates.

### Binding efficiency to insoluble cellulose

The molar absorption coefficients calculated for native and chimeric Dtur CelA (88500 M^−1^cm^−1^ and 115800 M^−1^cm^−1^) are in good agreement with those predicted from the tryptophan and tyrosine contents (Dtur CelA 88760 M^−1^cm^−1^, chimeric Dtur CelA 135700 M^−1^cm^−1^). The ability of native and modified Dtur CelA to bind microcrystalline cellulose was tested on Avicel at different time periods after protein purification and the time variable did not produce negative effects on both proteins (data not shown). The binding isotherms were regressed by graphing a double reciprocal plot of bound versus free protein (native or chimeric variant) (Figures S11, S12 of supplementary material) and the affinity constants were calculated. A linear plot was obtained, indicating a single type of specific interaction. Both the enzymes demonstrated affinity to insoluble cellulose, with higher values registered for chimeric Dtur CelA. Native Dtur CelA showed an association constant (K_a_) of 1.01 µM^−1^, while the chimeric variant showed a three times higher K_a_ of 3.28 µM^−1^. The K_a_ obtained for chimeric Dtur CelA towards Avicel was comparable to those observed for the *C*. *thermocellum* CBM3a (2.5 µM^−1^) of the scaffoldin subunit of the cellulosome^27^ and *C*. *cellulovorans* CbpA (1.67 µM^−1^)^28^. The binding efficiency of chimeric Dtur CelA towards the long chains of Avicel is consistent with the results of Viegas and collaborators^19^, who observed a higher affinity of *Ct*CBM11 for larger ligands when compared to those with the minimal length to fit the binding cleft. This difference is probably due to the presence of more contact points in larger oligosaccharides.

## Conclusions

This study reports for the first time the production of DturCelA as a chimeric protein. This fusion protein, while being still active on soluble glucans, shows higher activity on crystalline cellulose when compared to the native enzyme.

The addition of a carbohydrate binding module increased the enzyme’s stability at extreme pHs, making it usable in a broad range of process conditions. Moreover, its higher affinity to insoluble cellulose presents the economic advantage of reducing the enzyme loading to get effective hydrolysis rates. Despite the amount of literature reporting the fusion of different CBMs with GH5 enzymes, this GH family comprises so many enzymes from different species and with different properties that the resulting fusion proteins could show a different behavior from that predictable a priori. This study revealed important information about the interactions of the enzyme with its possible substrates. For a useful industrial application, the promising traits of this chimeric cellulase make it a good candidate for the degradation of lignocellulosic biomass, by employing it together with other cellulases for the production of enhanced enzymatic cocktails in the effort to improve the hydrolysis step in the biorefinery.

## Materials and Methods

### Gene cloning

Based on the published sequences of *D*. *turgidum CelA* and *C*. *thermocellum CelH* genes (GenBank Accession No. 7083332 and M31903.1, respectively), DNA sequences encoding Dtur CelA and the 48 aa linker peptide plus CBM11 from *Ct*Lic26A-Cel5E (CelH) were amplified with primers (Table 3) that allow their insertion into the pET20b vector (Novagen). The DNA fragments were purified using the Nucleo-Spin Extract kit (Macherey-Nagel, Germany). The catalytic module and the linker peptide plus CBM11 of the wild type vectors were combined by PCR amplification to produce a chimeric enzyme. PCR was also used for insertion of restriction sites and preservation of reading frames in the new constructs. The *Ct*CBM11 module together with its adjacent linker was fused to the C-terminus of Dtur CelA catalytic module. All constructs were designed to contain a C-terminal His tag for subsequent purification steps. *Escherichia coli* clones containing the plasmids pET28a-CelA and pET28a-CelH were provided by C5–6 Technologies (WI, USA).

**Table 3:** List of primers for the wild-type and chimeric protein used in this study.

Construct	Module	Primer name	Nucleotide sequence (5′-3′)
Dtur CelA	*Dtur*CelA	CelADtur-NdeI-F	ACTGCATATGAACAATCTTCCTATTAAGAGA
		CelADturHis_6_S-XhoI-R	CAGTCTCGAGTCAATGGTGATGGTGATGGTGTATATTCCTTTCAGGTATTAA
Chimeric Dtur CelA	*Dtur*CelA	CelADtur2-NdeI-F	ACTGCATATGAACAATCTTCCTATTAAGAG
		CelADturNS-HindIII-R	CAGTAAGCTTTATATTCCTTTCAGGTATTAATG
	*Cther*CelH Carbohydrate binding module	CelHCBM11-HindIII-F	ACTGAAGCTTGACATGGCTATTTACAACAGAG
		CelHCBM11His_6_S-XhoI-R	CAGTCTCGAGTCAATGGTGATGGTGATGGTGTCCGTGTTT TATTGAAGG

Restriction sites of NdeI, HindIII and XhoI are underlined.

### Protein expression and purification

*E*. *coli* BL21(DE3) cells (Stratagene, Canada) were transformed with plasmids pET-CelA-His_6_ and pET-CelA-linkerpep-CBM11-His_6_ and grown at 37 °C in Luria-Bertani medium containing 50 μg mL^−1^ ampicillin. At A_600_ of 0.5, IPTG was added to a final concentration of 0.4 mM and cultures were incubated at 37 °C for 3 h. Cells were harvested by centrifugation (4,000 g, 30 min, 4 °C). Proteins were purified under native conditions using the ‘Ni-NTA Agarose’ resin (QIAGEN, Germany) following the protocol recommended by the manufacturing company. Briefly, cell pellets were resuspended in lysis buffer (50 mM NaH_2_PO_4_, pH 8.0, 300 mM NaCl, 10 mM imidazole) and sonicated. The mixture was centrifuged (20,000 g, 30 min, 4 °C) to remove the cell debris. One mL bed volume Ni-nitrilotriacetic acid (NTA) Agarose resin was poured in a Poly-Prep^®^ chromatograhy column (Bio-Rad, USA) and equilibrated in lysis buffer. Then the supernatant was mixed with the resin for 1 h on a rotator at 4 °C. The column was washed twice with 4 volumes of wash buffer (50 mM NaH_2_PO_4_, pH 8.0, 300 mM NaCl, 20 mM imidazole). Elution was performed by using 50 mM NaH_2_PO_4_, pH 8.0, 300 mM NaCl, 250 mM imidazole. The purity and molecular mass of recombinant proteins were checked by SDS-PAGE, using Precision Plus Protein standard (Biorad) for molecular weight estimation. The gel images were acquired by the use of a densitometer (Image Scanner III, GE Healthcare, Sweden) at a resolution of 300 dpi. The molecular weights of Dtur CelA and chimeric Dtur CelA were calculated from their primary structure deduced from DNA sequence and compared with the experimental ones. Protein-containing fractions were pooled and dialyzed overnight at 4 °C against storage buffer (50 mM Tris-HCl, pH 7.5, 100 mM NaCl). Then, 20% glycerol was added to the dialyzed fractions and they were stored at 4 °C until use. Proteins were quantified by the method of Bradford with BSA as the protein standard^29^.

### Two dimensional electrophoresis (2-DE)

The protein samples were mixed with rehydration buffer (7 M Urea, 2 M Thiourea, 4% w/v CHAPS, 50 mM DTT, 5% Triton X100, 5% 4–7 IPG Buffer (GE Healthcare, Sweden), and traces of bromophenol blue (BBF)). First dimension isoelectric focusing (IEF) was performed using 7 cm immobilized linear pH range 4–7 strips on an IPG-Phor unit (GE Healthcare, Sweden). After 7 h of passive rehydration, IEF was performed at 20 °C under the following conditions: 30 V for 7 h, 300 V for 4 h, a gradient step to 1000 V for 30 min, a gradient step to 5000 V for 1 h 30 min and a holding step of 5000 V for a total of 8000 V hours. Immediately before the second dimension, the focused strips were first reduced for 15 min in SDS equilibration buffer (50 mM Tris-HCl pH 8.8, 6 M Urea, 30% v/v Glycerol, 2% w/v SDS) containing DTT (65 mM), and then alkylated for 15 min in SDS equilibration buffer with iodoacetamide (243 mM). Equilibrated strips were placed onto 10 × 8 cm vertical 12% (Dtur CelA) or 10% (chimeric Dtur CelA) polyacrylamide gel. SDS-PAGE was performed with a Mini Protean II Xi System (Bio-Rad, USA). The samples were run at 10 mA per gel for 30 min and then at 30 mA per gel until the tracking dye front reached the bottom of the gel. After SDS-separation, 2DE gels were fixed in a solution containing 40% methanol and 10% acetic acid and stained overnight with Colloidal Coomassie Brilliant Blue G250 (Bio-Rad, CA, USA)^30^. The images of the gels were acquired as indicated above.

### In-gel digestion

The protein spots were manually excised from 2DE gels, placed into 1.5 mL tubes previously cleaned with 50% acetonitrile (ACN) in HPLC-water, and destained overnight with 50% methanol and 5% acetic acid. The gel pieces were shrunk with 100% ACN, dried in a SpeedVac concentrator 5301 (Eppendorf, Germany) and rehydrated with 100 mM ammonium bicarbonate (NH_4_HCO_3_) for 15 min. Then an equal volume of ACN was added for 10 min, followed by drying. The gel pieces were rehydrated with 100 mM NH_4_HCO_3_ for 10 min and dried again. Trypsin (Sequencing Grade, Roche, Germany) was reconstituted in 50 mM NH_4_HCO_3_ at a concentration of 25 ng/µL and added to the gel pieces. The digestion was performed overnight at 37 °C. The supernatant was collected in a new vial and peptide extraction was carried out twice in 50% ACN/0.1% formic acid (FA) for 10 min with ultrasonication. The supernatants were pooled, dried and stored at −20 °C until mass spectrometry (MS) analysis.

### Protein identification by nanoLC coupled with Q TOF MS/MS

All nano-HPLC MS/MS experiments were performed on a Q-Star XL (Applied Biosystems, USA) connected to an Ultimate 3000 system equipped with a WPS-3000 autosampler and two low-pressure gradient micropumps LPG-3600 (LC Packings, NL), as described by Lingua *et al*.^31^. The Q-STAR XL operated in positive mode and information-dependent acquisition (IDA) mode, the dynamic exclusion feature of the Analyst QS 1.1 software (Applied Biosystems, CA, USA) was enabled, with an exclusion mass width of 3 m/z for 60 s. LC-MS/MS files obtained from each protein sample were merged into a single MASCOT generic format file (.mgf) and searched against the NCBI non-redundant database. Carbamidomethylation of cysteine residues, oxidation of methionine, deamidation of asparagine and glutamine were set as variable modifications for all Mascot searches. One missed trypsin cleavage site was allowed; tolerance for precursor and fragment masses was 0.25 Da.

### Zymography

In-gel endo-β−1,4-glucanase activity of Dtur CelA and chimeric Dtur CelA was detected on 10% polyacrylamide gels containing 0.4% AZO-carboxymethyl cellulose (AZO-CMC, Sigma-Aldrich, MO, USA). Proteins were mixed with an equal volume of 2 × non-reducing Laemmli buffer (2% w/v SDS, 20% Glycerol, 125 mM Tris-HCl pH 6.8). The gels were run at 30 mA and washed four times with 50 mM sodium-acetate buffer pH 5.8 for 10 min at room temperature. Enzymatic activity was observed as a clear zone contrasting on a dark blue background. The images were acquired as described before.

### Enzyme characteristics

The optimal pH and temperature of native and chimeric Dtur CelA were determined on 1% low viscosity barley β-glucan (Megazyme, Ireland) using the Nelson-Somogyi method for reducing sugars^32,33^. For determining the optimal pH, 50 mM sodium-acetate (pH 3.8-5.8) or 50 mM Tris-HCl (pH 6.8–8.8) buffers were used. All subsequent enzyme assays were performed at the optimal parameters unless otherwise noted. Different concentrations of barley β-glucan, ranging from 0.0015 mM to 0.025 mM in 50 mM sodium acetate, were used to determine the Michaelis-Menten constant (K_m_) and the maximum reaction velocity (V_max_). The Hanes-Woolf plot was used to infer the K_m_ and V_max_ of the two enzymes. The catalytic (K_cat_) and specificity (K_cat_/Km) constants were calculated as well.

### Enzyme assays

Enzyme activity on β-linked soluble polysaccharides was tested on 1% barley β-glucan using the reducing sugar assay mentioned above. The endo-glucanase specificity was determined on insoluble azurine-cross-linked-hydroxyethyl cellulose (AZCL-HE-Cellulose) (Cellazyme C tablets, Megazyme, Ireland) and the absorbance of the soluble dyed fragments was read at 590 nm. Enzymatic hydrolysis of insoluble cellulose was assayed using Avicel PH-101 (Sigma-Aldrich, USA) as substrate, following the method described by Reyes-Ortiz *et al*.^25^ with some modifications. The reaction mixture contained 10 mg of Avicel mixed with an aliquot of the enzyme plus β-glucosidase (C5–6 Technologies, USA) in a 1:1 ratio in a final volume of 500 µL of 50 mM sodium acetate. After incubation at 50 °C for 24 h in a thermo shaker, the substrate was removed by centrifugation and reducing sugar formation was determined using the dinitrosalicylic acid reagent (DNS) with a glucose standard curve^34^. Blanks without the enzyme were also included following the same procedure. Enzyme stability at 4 °C was also assessed by measuring activity over time (nine months). The samples were analyzed at least in triplicate and mean values were calculated. Enzyme activity is expressed as μmol of reducing sugars equivalent produced per minute per 1 µmol enzyme. Statistical analysis was performed with StatView 4.5 (Abacus Concepts, NJ, USA). Differences between the values were assessed using the F-test: P < 0.05 was considered to be significant.

### Measurement of native and chimeric Dtur CelA affinity to cellulose

The ability of Dtur CelA and its chimeric variant to bind micro-crystalline cellulose was tested on Avicel PH-101 (20 mg in 50 mM sodium-phosphate, pH 7.0), as described by Lee *et al*.^35^. The unbound proteins in the supernatant were recovered after 1 h of incubation and removal of the insoluble cellulose together with adsorbed proteins by centrifugation at 20,000 g for 5 min. Centrifugation of supernatants was repeated twice to ensure removal of particulate material. The concentration of unbound protein was determined by reading the absorbance at 280 nm using a DU800 spectrophotometer (Beckman Coulter, CA, USA). The amount of bound protein was deduced subtracting the amount of unbound protein from initial protein concentration. Multiple replicates of samples (at least six) were analyzed and mean values were calculated. The Langmuir isotherm was used to evaluate the binding of the two enzymes to Avicel. An isotherm of bound (µmol/g Avicel) vs free (µM) protein was generated and binding parameters were determined by graphing a double-reciprocal equation (1) (see below) as previously described^36^:

1 $$\,\frac{1}{B}=\frac{1}{{N}_{0}{K}_{a}}\times \frac{1}{F}+\frac{1}{{N}_{0}}$$

where F is the equilibrium concentration of unbound protein, B is the equilibrium concentration of bound protein, K_a_ is the equilibrium binding constant (µM^−1^) and N_0_ is the equilibrium concentration of available binding sites on Avicel (µmol/g Avicel).

Molar absorption coefficients were experimentally calculated for each fusion protein using the Lambert-Beer law and compared with the theoretical values obtained with the web tool Peptide Property Calculator (http://www.basic.northwestern.edu/biotools/proteincalc.html), which assumes that the spectral contributions of the tyrosine, tryptophan and cystine at 280 nm do not differ significantly in the native form of the protein, relatively to the denatured form, and that the protein contains no other chromophores^37^.

### Data availability statement

The datasets used and/or analysed during the current study are available from the corresponding author on reasonable request.

## Electronic supplementary material

Supplementary materials
