# Supplementary material for: Enhanced features of Dictyoglomus turgidum Cellulase A engineered with carbohydrate binding module 11 from Clostridium thermocellum
Source: Sci Rep. 2018 Mar 13;8:4402. doi: 10.1038/s41598-018-22769-w (PMC5849603; doi:10.1038/s41598-018-22769-w)
Supplement: Supplementary file 1 — Supplementary materials [file 41598_2018_22769_MOESM1_ESM.pdf]

Enhanced features of *Dictyoglomus turgidum* Cellulase A engineered with carbohydrate binding module 11 from *Clostridium thermocellum*.

Chiara Cattaneo[1*], Patrizia Cesaro[2*], Stefano Spertino[2], Sara Icardi[2], Maria Cavaletto[1,2]

University of Piemonte Orientale, Dipartimento di Scienze e Innovazione Tecnologica
[1]Complesso Universitario S. Giuseppe, Piazza S. Eusebio 5 – Vercelli 13100 Italy
[2]Viale T. Michel 11 –Alessandria 15121 Italy

Supplementary file 1: SDS-PAGE of native (left) and chimeric (right) Dtur CelA after purification by affinity chromatography. Lane 1: Molecular weight markers; lane 2: total protein extract; lane 3: flow through; lanes 4-7: elution fractions. The gels were Coomassie stained.

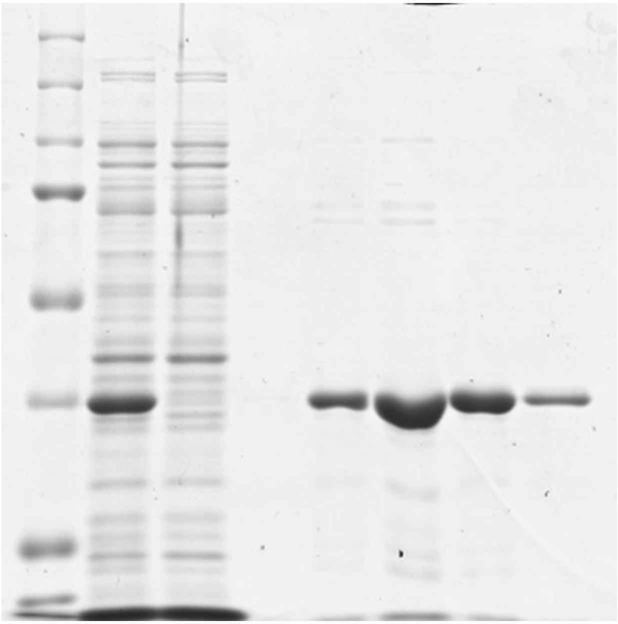

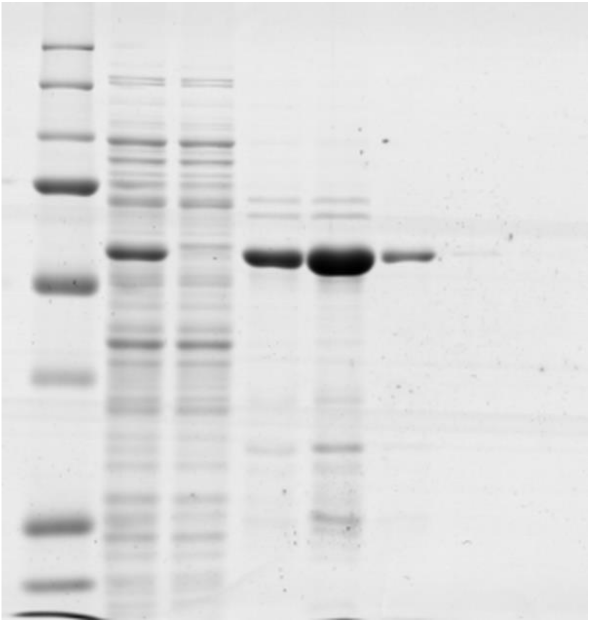

Supplementary file 2: MS sequence of spot 1 (Dtur CelA)

```
  1 MNNLPIKRGI NFGDALEAPY EGAWSGYIIK DEYFKIVKDA GFDHVRIPIK
 51 WSVYTQKEAP YSIEKRIFDR VDHLIEEGLK NNLHVIINIH HYEEIMEDPL
101 GEKERFLAIW RQISEHYKDY PNNLYFELLN EPTQNLSSEL WNQFLKEAIE
151 VIRRTNPERK IIVGPDNWNS LYNLEKLIIP ENDENIIITF HYYNPFPFTH
201 QGAGWVKIDL PVGVKWLGTE EEKREIEREL DMAVSWAEEH GNIPLYMGEF
251 GAYSKADMES RVRWTDFVAR SAEKRGIAWS YWEFYSGFGV FDPEKNEWRT
301 PLLRALIPER NI
```

Supplementary file 3: MS sequence of spot 2 (Dtur CelA)

```
  1 MNNLPIKRGI NFGDALEAPY EGAWSGYIIK DEYFKIVKDA GFDHVRIPIK
 51 WSVYTQKEAP YSIEKRIFDR VDHLIEEGLK NNLHVIINIH HYEEIMEDPL
101 GEKERFLAIW RQISEHYKDY PNNLYFELLN EPTQNLSSEL WNQFLKEAIE
151 VIRRTNPERK IIVGPDNWNS LYNLEKLIIP ENDENIIITF HYYNPFPFTH
201 QGAGWVKIDL PVGVKWLGTE EEKREIEREL DMAVSWAEEH GNIPLYMGEF
251 GAYSKADMES RVRWTDFVAR SAEKRGIAWS YWEFYSGFGV FDPEKNEWRT
301 PLLRALIPER NI
```

Peptides identified by LC-MS/MS analysis are in bold red

Supplementary file 4: MS sequence of spot 3 (Chimeric Dtur CelA)

Glycoside hydrolase 5 from *D. turgidum*

1 MNNLPIKR**GI** **NFGDALEAPY** **EGAWSGYIIK** **DEYFK**IVK**DA** **GFDHVRIPIK**
51 **WSVYTQK**EAP YSIEKR**IFDR** **VDHLIEEGLK** NNLHVIINIH HYEEIMEDPL
101 GEKERFLAIW RQISEHYKDY PNNLYFELLN EPTQNLSSEL WNQFLKEAIE
151 VIRRTNPER**K** **IIVGPDNWNS** **LYNLEK**LIIP ENDENIIITF HYYNPFPFTH
201 QGAGWVK**IDL** **PVGVK**WLGTE EEKREIER**EL** **DMAVSWAEEH** **GNIPLYMGEF**
251 **GAYSK**ADMES RVRWTDFVAR SAEKRGIAWS YWEFYSGFGV FDPEKNEWRT
301 PLLRALIPER NI

Carbohydrate-binding family 11 protein of Cel H from *C. thermocellum*

1 MKKRLLVSFL VLSIIVGLLS FQSLGNYNSG LKIGAWVGTQ PSESAIKSFQ
51 ELQGRKLDIV HQFINWSTDF SWVRPYADAV YNNGSILMIT WEPWEYNTVD
101 IKNGKADAYI TRMAQDMKAY GKEIWLRPLH EANGDWYPWA IGYSSRVNTN
151 ETYIAAFRHI VDIFRANGAT NVKWVFNVNC DNVGNGTSYL GHYPGDNYVD
201 YTSIDGYNWG TTQSWGSQWQ SFDQVFSRAY QALASINKPI IIAEFASAEI
251 GGNKARWITE AYNSIRTSYN KVIAAVWFHE NKETDWRINS SPEALAAYRE
301 AIGAGSSNPT PTPTWTSTPP SSSPKAVDPF EMVRKMGMGT NLGNTLEAPY
351 EGSWSKSAME YYFDDFKAAG YKNVRIPVRW DNHTMRTYPY TIDKAFLDRV
401 EQVVDWSLSR GFVTIINSHH DDWIKEDYNG NIERFEKIWE QIAERFKNKS
451 ENLLFEIMNE PFGNITDEQI DDMNSRILKI IRKTNPTRIV IIGGGYWNSY
501 NTLVNIKIPD DPYLIGTFHY YDPYEFTHKW RGTWGTQEDM DTVVRVFDFV
551 KSWSDRNNIP VYFGEFAVMA YADRTSRVKW YDFISDAALE RGFACSVWDN
601 GVFGSLDNDM AIYNRDTRTF DTEILNALFN PGTYPSYSPK PSPTPRPTKP
651 PVTPAVGEK**M** **LDDFEGVLNW** **GSYSGEGAK**V STKIVSGKTG NGMEVSYTGT
701 TDGYWGTVYS LPDGDWSKWL KISFDIKSVD GSANEIRFMI AEK**SINGVGD**
751 **GEHWVYSITP** **DSSWKTIEIP** **FSSFR**RRLDY QPPGQDMSGT LDLDNIDSIH
801 FMYANNKSGK **FVVDNIKLIG** **ATSDPTPSIK** HGDLNFDNAV NSTDLLMLKR
851 YILKSLELGT SEQEEKFKKA ADLNRDNKVD STDLTILKRY LLKAISEIPI
901

Peptides identified by LC-MS/MS analysis are in bold red

Supplementary file 5: MS sequence of spot 4 (Chimeric Dtur CelA)

Glycoside hydrolase 5 from *D. turgidum*

1 **MNNLPIKRGI NFGDALEAPY EGAWSGYIIK DEYFKIVKDA GFDHVRIPIK**
51 **WSVYTQKEAP YSIEKRIFDR VDHLIEEGLK NNLHVIINIH HYEEIMEDPL**
101 **GEKER**FLAIW R**QISEHYKDY PNNLYFELLN EPTQNLSSEL WNQFLKEAIE**
151 **VIRR**TNPER**K IIVGPDNWNS LYNLEKLIIP ENDENIIITF HYYNPFPFTH**
201 **QGAGWVKIDL PVGVKWLGTE EEKR**EIER**EL DMAVSWAEEH GNIPLYMGEF**
251 **GAYSK**ADMES R**VRWTDFVAR** SAEKR**GIAWS YWEFYSGFGV FDPEKNEWR**T
301 PLLRALIPER NI

Carbohydrate-binding family 11 protein of Cel H from
C. thermocellum

1 MKKRLLVSFL VLSIIVGLLS FQSLGNYNSG LKIGAWVGTQ PSESAIKSFQ
51 ELQGRKLDIV HQFINWSTDF SWVRPYADAV YNNGSILMIT WEPWEYNTVD
101 IKNGKADAYI TRMAQDMKAY GKEIWLRPLH EANGDWYPWA IGYSSRVNTN
151 ETYIAAFRHI VDIFRANGAT NVKWVFNVNC DNVGNGTSYL GHYPGDNYVD
201 YTSIDGYNWG TTQSWGSQWQ SFDQVFSRAY QALASINKPI IIAEFASAEI
251 GGNKARWITE AYNSIRTSYN KVIAAVWFHE NKETDWRINS SPEALAAYRE
301 AIGAGSSNPT PTPTWTSTPP SSSPKAVDPF EMVRKMGMGT NLGNTLEAPY
351 EGSWSKSAME YYFDDFKAAG YKNVRIPVRW DNHTMRTYPY TIDKAFLDRV
401 EQVVDWSLSR GFVTIINSHH DDWIKEDYNG NIERFEKIWE QIAERFKNKS
451 ENLLFEIMNE PFGNITDEQI DDMNSRILKI IRKTNPTRIV IIGGGYWNSY
501 NTLVNIKIPD DPYLIGTFHY YDPYEFTHKW RGTWGTQEDM DTVVRVFDFV
551 KSWSDRNNIP VYFGEFAVMA YADRTSRVKW YDFISDAALE RGFACSVWDN
601 GVFGSLDNDM AIYNRDTR**TF DTEILNALFN PGTYPSYSPK PSPTPRPTKP**
651 **PVTPAVGEKM LDDFEGVLNW GSYSGEGAK**V STKIVSGK**TG NGMEVSYTGT**
701 **TDGYWGTVYS LPDGDWSK**WL K**ISFDIK**SVD GSANEIR**FMI AEKSINGVGD**
751 **GEHWVYSITP DSSWKTIEIP FSSFRRRLDY QPPGQDMSGT LDLDNIDSIH**
801 **FMYANNK**SGK **FVVDNIKLIG ATSDPTPSIK** HGDLNFDNAV NSTDLLMLKR
851 YILKSLELGT SEQEEKFKKA ADLNRDNKVD STDLTILKRY LLKAISEIPI
901

Peptides identified by LC-MS/MS analysis are in bold red

Supplementary file 6: MS sequence of spot 5 (Chimeric Dtur CelA)

Glycoside hydrolase 5 from *D. turgidum*

1 **MNNLPIKRGI NFGDALEAPY EGAWSGYIIK DEYFKIVKDA GFDHVRIPIK**
51 **WSVYTQKEAP YSIEKRIFDR VDHLIEEGLK NNLHVIINIH HYEEIMEDPL**
101 **GEKER**FLAIW RQISEHYKDY PNNLYFELLN EPTQNLSSEL WNQFLK**EAIE**
151 **VIR**RTNPER**K IIVGPDNWNS LYNLEKLIIP ENDENIIITF HYYNPFPFTH**
201 **QGAGWVKIDL PVGVKWLGTE EEKR**EIER**EL DMAVSWAEEH GNIPLYMGEF**
251 **GAYSK**ADMES RVR**WTDFVAR** SAEKR**GIAWS YWEFYSGFGV FDPEKNEWR**T
301 PLLRALIPER NI

Carbohydrate-binding family 11 protein of Cel H from *C. thermocellum*

1 MKKRLLVSFL VLSIIVGLLS FQSLGNYNSG LKIGAWVGTQ PSESAIKSFQ
51 ELQGRKLDIV HQFINWSTDF SWVRPYADAV YNNGSILMIT WEPWEYNTVD
101 IKNGKADAYI TRMAQDMKAY GKEIWLRPLH EANGDWYPWA IGYSSRVNTN
151 ETYIAAFRHI VDIFRANGAT NVKWVFNVNC DNVGNGTSYL GHYPGDNYVD
201 YTSIDGYNWG TTQSWGSQWQ SFDQVFSRAY QALASINKPI IIAEFASAEI
251 GGNKARWITE AYNSIRTSYN KVIAAVWFHE NKETDWRINS SPEALAAYRE
301 AIGAGSSNPT PTPTWTSTPP SSSPKAVDPF EMVRKMGMGT NLGNTLEAPY
351 EGSWSKSAME YYFDDFKAAG YKNVRIPVRW DNHTMRTYPY TIDKAFLDRV
401 EQVVDWSLSR GFVTIINSHH DDWIKEDYNG NIERFEKIWE QIAERFKNKS
451 ENLLFEIMNE PFGNITDEQI DDMNSRILKI IRKTNPTRIV IIGGGYWNSY
501 NTLVNIKIPD DPYLIGTFHY YDPYEFTHKW RGTWGTQEDM DTVVRVFDFV
551 KSWSDRNNIP VYFGEFAVMA YADRTSRVKW YDFISDAALE RGFACSVWDN
601 GVFGSLDNDM AIYNRDTR**TF DTEILNALFN PGTYPSYSPK PSPTPRPTKP**
651 **PVTPAVGEKM LDDFEGVLNW GSYSGEGAK**V STKIVSGKTG NGMEVSYTGT
701 TDGYWGTVYS LPDGDWSKWL K**ISFDIKSVD GSANEIRFMI AEKSINGVGD**
751 **GEHWVYSITP DSSWKTIEIP FSSFR**R**RLDY QPPGQDMSGT LDLDNIDSIH**
801 **FMYANNK**SGK **FVVDNIKLIG ATSDPTPSIK** HGDLNFDNAV NSTDLLMLKR
851 YILKSLELGT SEQEEKFKKA ADLNRDNKVD STDLTILKRY LLKAISEIPI
901

Peptides identified by LC-MS/MS analysis are in bold red

Supplementary file 7: MS sequence of spot 6 (Chimeric Dtur CelA)

Glycoside hydrolase 5 from *D. turgidum*

1 MNNLPIKR**GI NFGDALEAPY EGAWSGYIIK DEYFK**IVKDA GFDHVRIPIK

51 **WSVYTQK**EAP YSIEKR**IFDR VDHLIEEGLK** NNLHVIINIH HYEEIMEDPL

101 GEKERFLAIW RQISEHYKDY PNNLYFELLN EPTQNLSSEL WNQFLK**EAIE**

151 **VIR**RTNPERK **IIVGPDNWNS LYNLEKLIIP ENDENIIITF HYYNPFPFTH**

201 **QGAGWVKIDL PVGVK**WLGTE EEKREIEREL DMAVSWAEEH GNIPLYMGEF

251 GAYSKADMES RVR**WTDFVAR** SAEKR**GIAWS YWEFYSGFGV FDPEKNEWR**T

301 PLLRALIPER NI

Carbohydrate-binding family 11 protein of Cel H from *C. thermocellum*

1 MKKRLLVSFL VLSIIVGLLS FQSLGNYNSG LKIGAWVGTQ PSESAIKSFQ

51 ELQGRKLDIV HQFINWSTDF SWVRPYADAV YNNGSILMIT WEPWEYNTVD

101 IKNGKADAYI TRMAQDMKAY GKEIWLRPLH EANGDWYPWA IGYSSRVNTN

151 ETYIAAFRHI VDIFRANGAT NVKWVFNVNC DNVGNGTSYL GHYPGDNYVD

201 YTSIDGYNWG TTQSWGSQWQ SFDQVFSRAY QALASINKPI IIAEFASAEI

251 GGNKARWITE AYNSIRTSYN KVIAAVWFHE NKETDWRINS SPEALAAYRE

301 AIGAGSSNPT PTPTWTSTPP SSSPKAVDPF EMVRKMGMGT NLGNTLEAPY

351 EGSWSKSAME YYFDDFKAAG YKNVRIPVRW DNHTMRTYPY TIDKAFLDRV

401 EQVVDWSLSR GFVTIINSHH DDWIKEDYNG NIERFEKIWE QIAERFKNKS

451 ENLLFEIMNE PFGNITDEQI DDMNSRILKI IRKTNPTRIV IIGGGYWNSY

501 NTLVNIKIPD DPYLIGTFHY YDPYEFTHKW RGTWGTQEDM DTVVRVFDFV

551 KSWSDRNNIP VYFGEFAVMA YADRTSRVKW YDFISDAALE RGFACSVWDN

601 GVFGSLDNDM AIYNRDTRTF DTEILNALFN PGTYPSYSPK PSPTPRPTKP

651 PVTPAVGEK**M LDDFEGVLNW GSYSGEGAK**V STKIVSGK**TG NGMEVSYTGT**

701 **TDGYWGTVYS LPDGDWSK**WL K**ISFDIK**SVD GSANEIR**FMI AEKSINGVGD**

751 **GEHWVYSITP DSSWKTIEIP FSSFR**RRLDY QPPGQDMSGT LDLDNIDSIH

801 FMYANNKSGK **FVVDNIKLIG ATSDPTPSIK** HGDLNFDNAV NSTDLLMLKR

851 YILKSLELGT SEQEEKFKKA ADLNRDNKVD STDLTILKRY LLKAISEIPI

901

Peptides identified by LC-MS/MS analysis are in bold red

Supplementary file 8: Zymography on 0.4% AZO-CMC of native (left) and chimeric (right) Dtur CelA. Lane 1: Molecular weight marker; lane 2: purified recombinant enzyme.

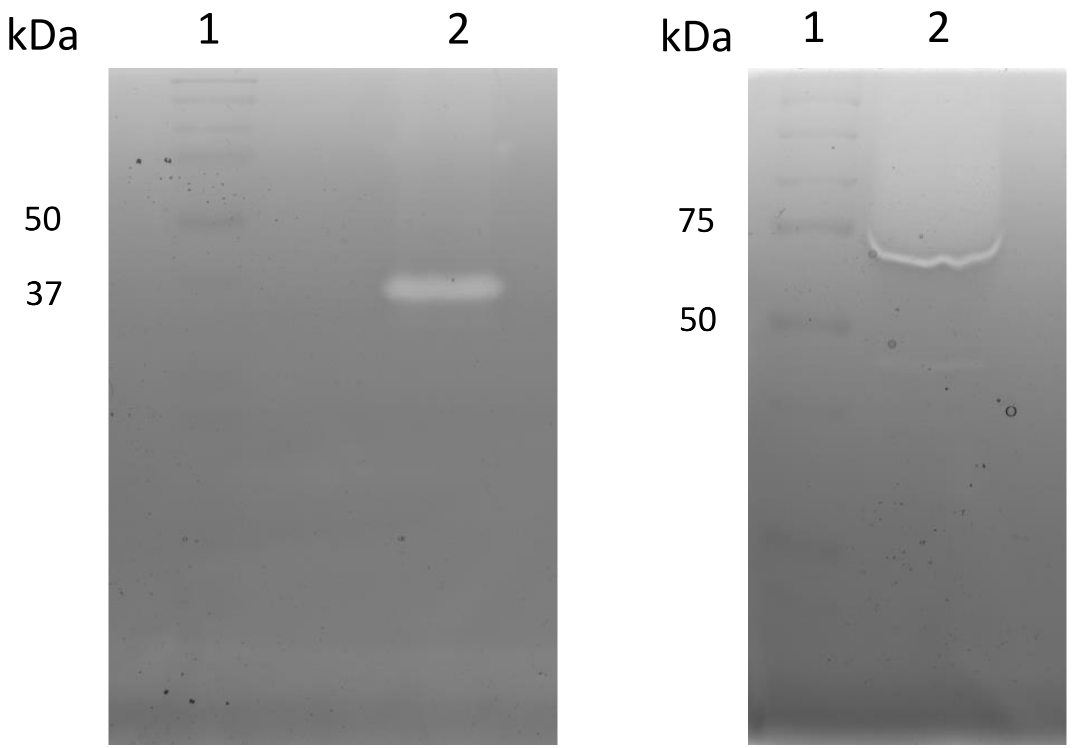

Supplementary file 9: Dtur CelA activity on barley beta-glucan vs time (days after protein production)

Enzyme	Time after production (days)	enzyme dilution (mg/ml)	Mean of enzyme activity (U/mg)	Standard Error	$p<0.05$
CelA	0	0.013	838,055	57,992	no
CelA	270	0.013	671,516	57,618	

Supplemenary file 10: Chimeric Dtur CelA activity on barley beta-glucan vs time (days after protein production)

Enzyme	Time after production (days)	enzyme dilution (mg/ml)	Mean of enzyme activity (U/mg)	Standard Error	$p<0.05$
chimeric-CelA	0	0.013	157,288	6,064	no
chimeric-CelA	270	0.013	136,571	9,07	

Supplementary file 11: Dtur CelA binding to Avicel

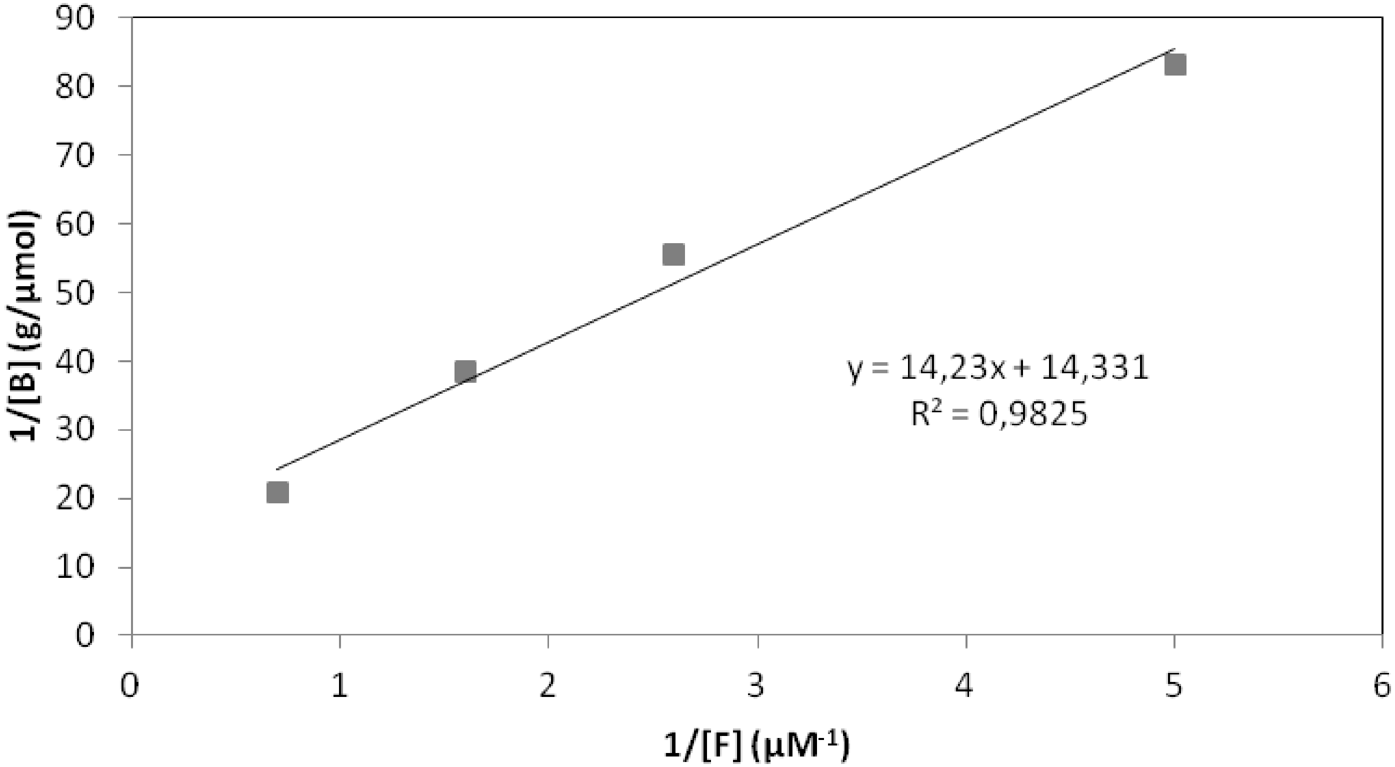

Double reciprocal plot of bound (B) versus free (F) protein.

Supplementary file 12: Chimeric Dtur CelA binding to Avicel

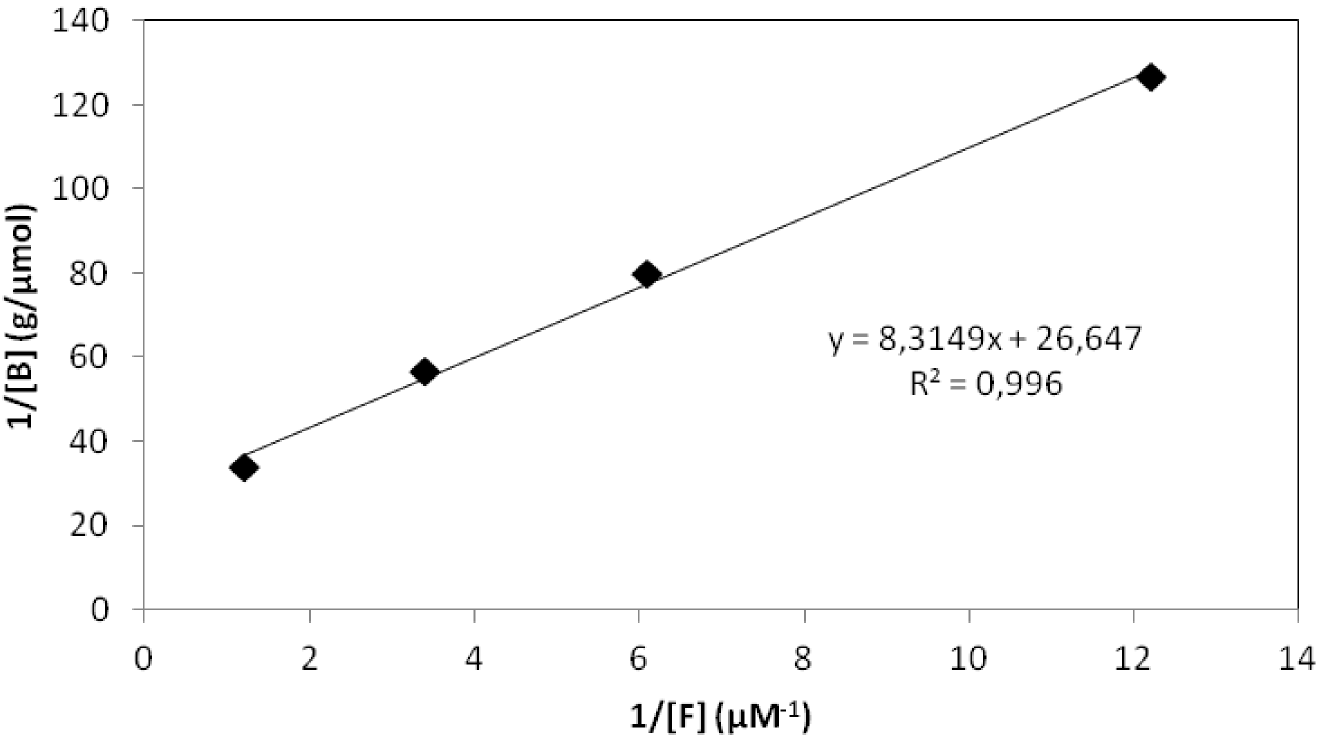

Double reciprocal plot of bound (B) versus free (F) protein.
